# Repeated bouts of resistance exercise with short recovery periods activates mTOR signaling, but not protein synthesis, in mouse skeletal muscle

**DOI:** 10.14814/phy2.13515

**Published:** 2017-11-28

**Authors:** Junya Takegaki, Riki Ogasawara, Yuki Tamura, Ryo Takagi, Yuki Arihara, Arata Tsutaki, Koichi Nakazato, Naokata Ishii

**Affiliations:** ^1^ Department of Life Sciences Graduate School of Arts and Sciences The University of Tokyo Tokyo Japan; ^2^ Department of Life Science and Applied Chemistry Nagoya Institute of Technology Nagoya Japan; ^3^ Graduate School of Health and Sport Science Nippon Sport Science University Tokyo Japan

**Keywords:** mTOR signaling, protein synthesis, recovery, resistance exercise

## Abstract

The recovery period between bouts of exercise is one of the major factors influencing the effects of resistance exercise, in addition to exercise intensity and volume. However, the effects of shortening the recovery time between bouts of resistance exercise on subsequent protein synthesis remain unclear. In this study, we investigated the consequences of shortening the recovery time between bouts of resistance exercise on protein synthesis and related processes in mouse skeletal muscles. Eighteen male C57BL/6J mice were randomly subjected to three bouts of resistance exercise with 72 (72H), 24 (24H), or 8 h (8H) of recovery periods between bouts. Resistance exercise, consisting of five sets of 3 s × 10 isometric contractions with 3 min rest between sets, was elicited on the right tibialis anterior muscle via percutaneous electrical stimulation on the deep peroneal nerve under isoflurane anesthesia. The left muscle served as an internal control. Six hours after the third bout of exercise, protein synthesis was found to be activated in the 72H and 24H groups, but not in the 8H group. Phosphorylation of p70S6K at Thr 389, a marker of mammalian target of rapamycin (mTOR) signaling, was increased in all groups, with the 8H group showing the highest magnitude. In contrast, protein carbonylation was observed only in mice in the 8H group. These results suggest that repeated bouts of resistance exercise with 8 h of recovery periods do not effectively increase the levels of muscle protein synthesis despite activation of the mTOR signaling pathway, which likely involves oxidative stress.

## Introduction

Resistance exercise is widely recognized as the most effective way to increase skeletal muscle mass and strength. Hypertrophic adaptation of the muscles develops as a result of the accumulation of anabolic responses to repeated exercise bouts with appropriate recovery (Tipton and Wolfe 2001; Ogasawara et al. 2016). A recovery period of 2 or 3 days is suitable for gaining effects following exercise in the same muscle (Kraemer and Ratamess 2004). A deficit of recovery leads to the accumulation of training stress, causing inflammatory or catabolic responses (Coffey et al. 2007; Souza et al. 2014). However, little is known about the effect of shortening the recovery between bouts on protein anabolic responses induced by resistance exercise.

The mammalian/mechanistic target of rapamycin (mTOR) signaling is a primary regulator of muscle translational activity and protein synthesis, and resistance exercise is known as one of the major activators of mTOR signaling (Baar and Esser 1999; Bodine et al. 2001; Nader et al. 2005; Drummond et al. 2009). One of the key factors influencing mTOR signaling is p70S6K, and the proportion of the phosphorylated form of p70S6K^Thr389^ is generally used as an indicator of mTOR signaling activity. Repetition of resistance exercise without a sufficient recovery period for p70S6K dephosphorylation has been shown to cause accumulation of the phosphorylated form of p70S6K^Thr389^ (Coffey et al. 2007). Therefore, repetition of resistance exercise with shorter recovery between bouts can induce stronger activation of mTOR signaling and translation. On the other hand, several factors are known to suppress protein translation and synthesis in response to excess translational activation.

Excess translation activation induces the accumulation of misfolded or unfolded proteins in the endoplasmic reticulum (ER) and causes ER stress, resulting in the unfolded protein response (Cao and Kaufman 2012). In the unfolded protein response, the phosphorylation of eukaryotic translation initiation factor 2*α* (eIF‐2*α*) downregulates translation initiation and suppresses protein synthesis (Kaufman 2004; Malhotra et al. 2008, 2008; Guan et al. 2014). Moreover, a previous study reported that a single session of resistance exercise triggered the unfolded protein response in human skeletal muscle (Ogborn et al., 2014). Therefore, several repetitions of resistance exercises with short recovery would highly activate mTOR signaling, a primary regulator of protein synthesis, but also may highly phosphorylate eIF‐2*α* and reduce protein synthesis following exercise. However, how the shortening of recovery between bouts affects protein synthesis following successive bouts of resistance exercise remains unclear.

This study investigated changes in the muscle anabolic effect corresponding to shortening of recovery periods between bouts of resistance exercises of equal volume and intensity by examining acute protein synthesis after three successive bouts of resistance exercise with varying recovery periods. We hypothesized that repetition of resistance exercise with recovery periods shorter than a certain limit does not effectively induce protein synthesis in the muscles, even though mTOR signaling is activated.

## Materials and Methods

### Animals

Male C57BL/6J mice at 10–11 weeks old (*n* = 18) were obtained from CLEA Japan (Tokyo, Japan). All animals were housed in an isolated and environmentally controlled room at 22 ± 2°C under a 12–12 h light–dark cycle and were provided with food and water ad libitum. The Ethics Committee for Animal Experiments at Nippon Sport Science University approved this study.

### Study design and resistance exercise protocol

Mice were randomly divided into three groups, which were subjected to resistance exercise every 72 (72H, *n* = 6), 24 (24H, *n* = 6), and 8 h (8H, *n* = 6). Under isoflurane anesthesia, the hair of the right hindlimb of each mouse was shaved off, and the shaved portion was cleaned with alcohol wipes. The foot of mouse was firmly attached to the foot plate (the ankle joint was fixed at 90°) in the supine position. Contraction of tibialis anterior muscle was induced via percutaneous stimulation of the deep peroneal nerve following the protocol described by Ambrosio et al. (2012). Resistance exercise and muscle sampling were carried out according to the protocol described by Ogasawara et al. (2016). Briefly, the right tibialis anterior muscle was exercised with five sets of isometric contractions at 3‐min rest intervals. Each set consisted of 10 contractions, with a 3‐sec duration per contraction and a 7‐sec interval in between contractions. The left tibialis anterior muscle served as an internal control. Stimulation frequency was set to 100 Hz, and the voltage was adjusted to produce maximal isometric torque. All mice completed three sessions of resistance exercise, and muscle samples were collected 6 h after the third session. Before the third session, mice were fasted overnight. Collected tissues were frozen at −80°C until use.

### Muscle protein synthesis

Muscle protein synthesis rates were measured according to the SUnSET method (Goodman et al. 2011). Briefly, a 0.04‐*μ*mol/g body weight puromycin solution was prepared using phosphate buffered saline and intraperitoneally injected into each mouse at 5 min after the beginning of anesthesia. Muscles were then excised at exactly 15 min after the puromycin injection. Following homogenization and centrifugation at 2000*g* for 3 min at 4°C, the supernatant was collected and processed for western blotting using antipuromycin antibody (Cat # MABE343, Merck Millipore).

### Western blotting

Muscle samples were homogenized in RIPA buffer (Thermo Fisher Scientific, USA) containing Halt™ protease and phosphatase inhibitor cocktail (Thermo Fisher Scientific, USA) and centrifuged at 10,000*g* for 10 min at 4°C, after which the supernatants were collected. The concentrations of the supernatants were determined using a protein concentration determination kit (DC™ Protein Assay kit, BioRad, USA). Samples were diluted in 3 ×  Blue Loading Buffer (Cell signaling, USA) and boiled at 95°C for 5 min. Proteins (25 *μ*g) were then subjected to 10% or 15% SDS‐polyacrylamide gel electrophoresis and subsequently transferred to polyvinylidene difluoride or nitrocellulose membranes. Membranes were blocked in 5% skim milk in Tris‐buffered saline‐Tween‐20 (TBST) for 1 h at room temperature and subsequently incubated overnight at 4°C with the following primary antibodies: phosphorylated (phospho)‐AMP‐activated protein kinase (AMPK, Thr172, #2531), total‐AMPK (#2532), phospho‐Akt (Ser473, #4051), total‐Akt (#9272), phospho‐mTOR (Ser2448, #2971), total‐mTOR (#2983), phospho‐p70S6K (Thr389, #9205), total‐p70S6K (#2708), phospho‐rpS6 (Ser240/244, #2215), total‐rpS6 (#2217), phospho‐JNK (Thr183/Tyr185, #9251), total‐JNK (#9252), phospho‐ERK (Thr202/Tyr204, #9101), total‐ERK (#9102), phospho‐4E‐BP1 (Thr37/46, #9459), total‐4E‐BP1 (#9452), phospho‐eIF2*α* (Ser51, #3597), and total‐eIF2*α* (#5324) purchased from Cell Signaling. Membranes were then incubated for 1 h at room temperature with the appropriate secondary antibodies. Chemiluminescent reagents (Clarity™ Western ECL Substrate, BioRad, USA) were used to visualize the protein bands. Images were scanned on a chemiluminescence detector (Ez‐Capture, ATTO, Japan). After scanning, membranes were stained with Coomassie Brilliant Blue (CBB) or Ponceau S to verify equal loading in all lanes and for normalization. Band intensities were quantified using Image J 1.46 software (National Institutes of Health, USA).

### Carbonylated protein

To evaluate levels of oxidative stress, we measured the amounts of carbonylated protein in the muscles using carbonylated protein detection kits following the manufacturer's instructions (Cosmo bio, Tokyo, Japan).

### Statistical analysis

All values were expressed as mean ± SEM. Data were analyzed using two‐way ANOVA (exercise × recovery. If an interaction was observed, Bonferroni multiple‐comparison testing was performed. Statistical significance was considered at *P* < 0.05.

## Results

### Muscle protein synthesis

**Figure 1:**
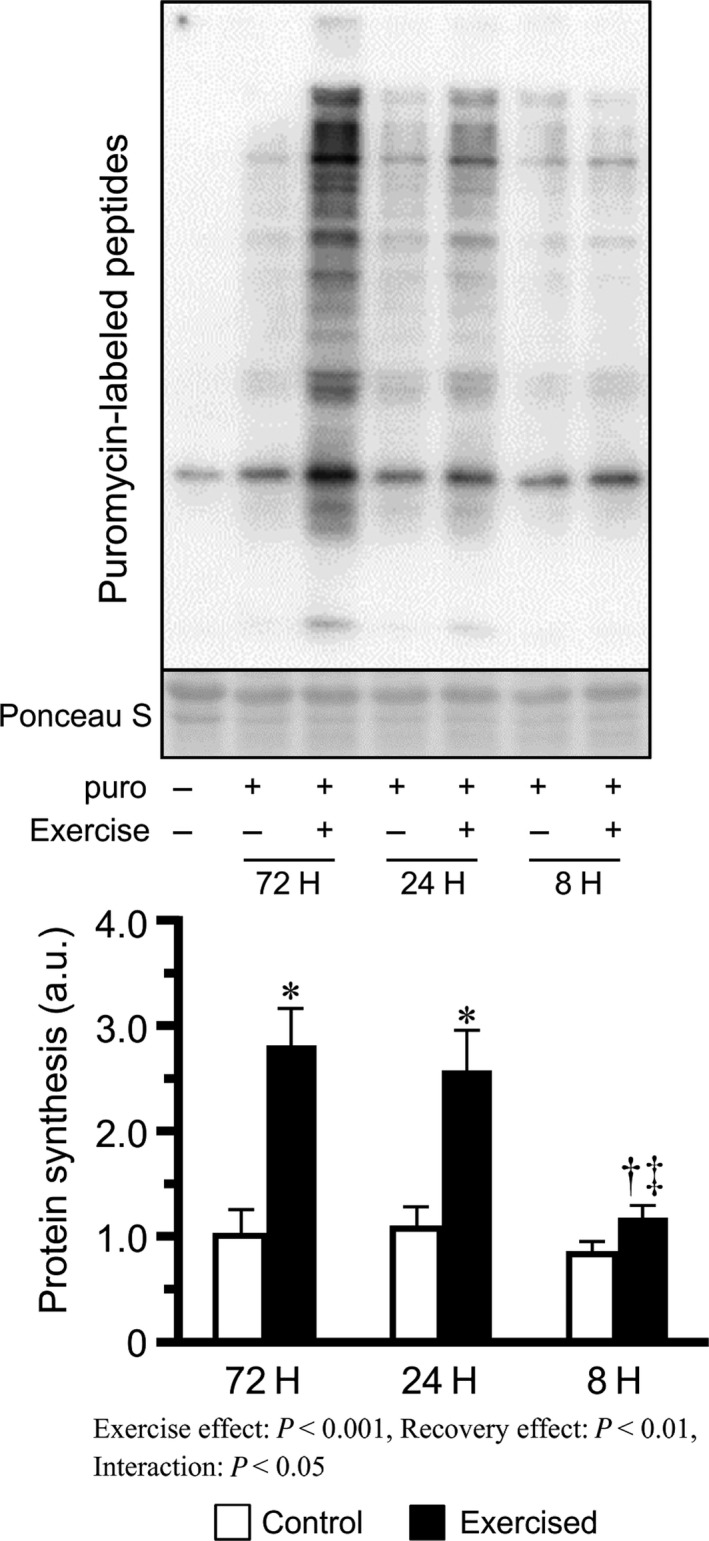
Protein synthesis after bouts of resistance exercise in mouse skeletal muscle. Values are expressed relative to the no exercise 72H group and presented as the mean + SE. *P <* *0.05 versus control in each group, ^†^ *P *<* *0.05 versus exercised muscle in the 72H group, ^‡^ *P *<* *0.05 versus exercised muscle in the 24H group.

Protein synthesis in exercised muscles was found to be elevated in the 72H and 24H groups, but not in the 8H group, when compared to the corresponding control muscles. In addition, protein synthesis in the exercised muscles of mice in the 8H group was significantly lower than those in the 72H and 24H groups (Fig. 1).

### Signaling proteins

**Figure 2:**
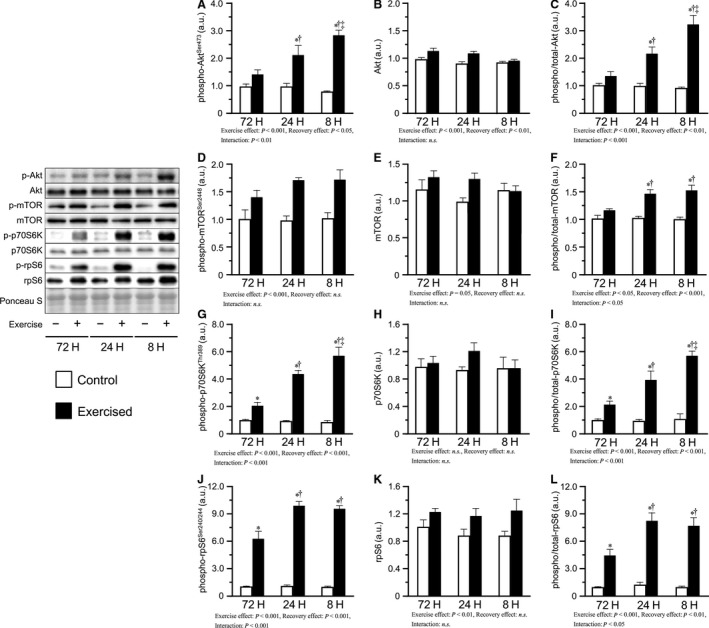
Phosphorylation and expression of proteins involved in mTOR signaling after bouts of resistance exercise in mouse skeletal muscle. Phosphorylated protein expression, total protein expression, and phosphorylated to total ratio of Akt (A–C), mTOR (D‐F), p70S6K (G–I), and rpS6 (J–L) after three bouts of resistance exercise. Values are expressed relative to the no exercise 72H group and presented as the mean + SE. *P <* *0.05 versus control in each group, ^†^ *P *<* *0.05 versus exercised muscle in the 72H group, ^‡^ *P *<* *0.05 versus exercised muscle in the 24H group. n.s. indicates not significant.

We measured the levels of phosphorylated Akt^Ser473^, mTOR^Ser2448^, p70S6K^Thr389^, and rpS6^Ser240/244^, which served as indicators of mTOR signaling. Bouts of resistance exercise increased phosphorylated Akt^Ser473^ and the phosphorylated/total ratio in the 24H and 8H groups, with the degree being increased as the recovery was shortened (Fig. 2A, C). Additionally, bouts of resistance exercise increased phosphorylated mTOR^Ser2448^ and the phosphorylated/total ratio in the 24H and 8H groups (Fig. 2D, F), but no differences were observed between the 24H and 8H groups. In contrast, bouts of resistance exercise increased phosphorylated p70S6K^Thr389^ and the phosphorylated/total ratio in all groups, with the degree increasing with the shortening of recovery (Fig. 2G, I). Additionally, phosphorylated rpS6^Ser240/244^ and the phosphorylated/total ratio were increased by bouts of resistance exercise, and the degree was higher in the shorter recovery groups, but no differences were observed between the 24H and 8H groups (Fig. 2J, L).

**Figure 3:**
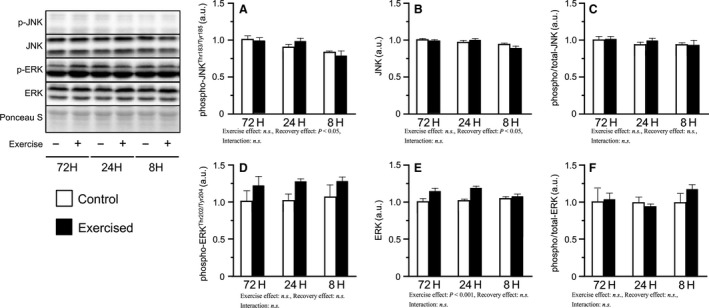
Phosphorylation and expression of JNK and ERK after bouts of resistance exercise in mouse skeletal muscle. Phosphorylated protein expression, total protein expression, and phosphorylated to total ratio of JNK (A–C) and ERK (D‐F) after three bouts of resistance exercise. Values are expressed relative to the no exercise 72H group and presented as the mean + SE. n.s. indicates not significant.

We next measured the levels of the phosphorylated forms of Jun‐N‐terminal kinase (JNK) and extracellular signal regulated kinase (ERK) 1/2, which contribute to the phosphorylation of p70S6K. Phosphorylated JNK^Thr183/Tyr185^ was slightly decreased in response to the shortening of recovery accompanied by a decrease in total‐JNK levels, whereas the phosphorylated/total ratio was not changed (Fig. 3A–C). Additionally, no changes were observed in the phosphorylated ERK1/2^Thr202/Tyr204^ and phosphorylated/total ratio (Fig. 3D, F).

**Figure 4:**
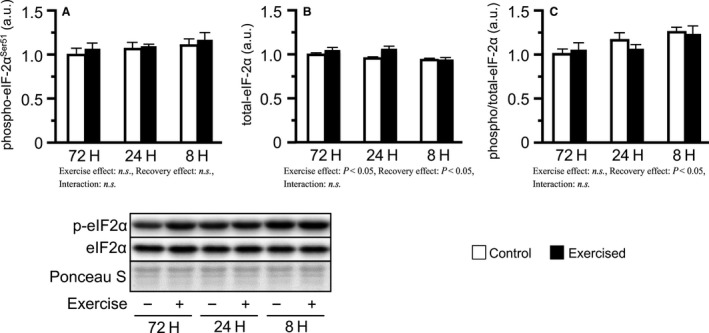
Phosphorylation and expression of eIF‐2*α* after bouts of resistance exercise in mouse skeletal muscle. Phosphorylated protein expression (A), total protein expression (B), and phosphorylated to total ratio (C). Values are expressed relative to the no exercise 72H group and presented as the mean + SE. n.s. indicates not significant.

**Figure 5:**
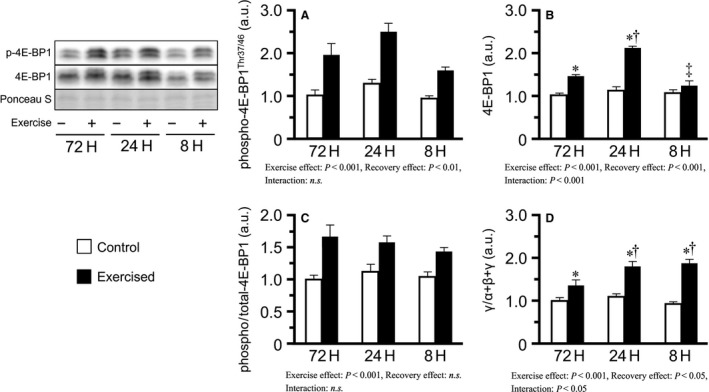
Phosphorylation and expression of 4E‐BP1 after bouts of resistance exercise in mouse skeletal muscle. Phosphorylated protein expression (A), total protein expression (B), phosphorylated to total ratio (C), and *γ* to total (*α * +  *β * +  *γ*) ratio (D) after three bouts of resistance exercise. Values are expressed relative to the no exercise 72H group and presented as the mean + SE. *P <* *0.05 versus control in each group, ^†^ *P *<* *0.05 versus exercised muscle in the 72H group, ^‡^ *P *<* *0.05 versus exercised muscle in the 24H group. n.s. indicates not significant.

Concerning the phosphorylation of translation initiation inhibiting factors, phosphorylated eIF‐2*α* ^Ser51^ levels did not change in any of the groups (Fig. 4A). Additionally, the phosphorylated/total ratio was slightly increased with shortened recovery (main effect of recovery), accompanied by a slight decrease in total eIF‐2*α* expression in the shorter groups (Fig. 4B, C). In contrast, bouts of resistance exercise increased the levels of phosphorylated 4E‐BP1^Thr37/46^ and the phosphorylated/total ratio (main effect of exercise), but no interaction was observed (Fig. 5A, C). Additionally, bouts of resistance exercise increased the active form ratio of 4E‐BP1 (*γ*/total *α*+*β*+*γ*), and the degree was higher in the 24H and 8H groups than in the 72H group (Fig. 5D).

**Figure 6:**
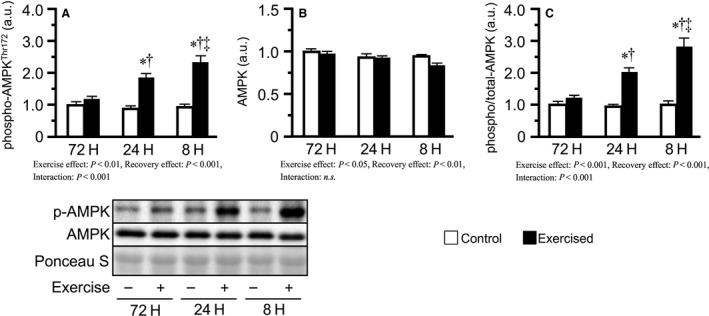
Phosphorylation and expression of AMPK after bouts of resistance exercise in mouse skeletal muscle. Phosphorylated protein expression (A), total protein expression (B), and phosphorylated to total ratio (C). Values are expressed relative to the no exercise 72H group and presented as the mean + SE. *P <* *0.05 versus control in each group, ^†^ *P *<* *0.05 versus exercised muscle in the 72H group, ^‡^ *P *<* *0.05 versus exercised muscle in the 24H group. n.s. indicates not significant.

In this study, we also measured AMPK as another factor suppressing protein synthesis. Bouts of resistance exercise increased phosphorylated AMPK^Thr172^ and the phosphorylated/total ratio in the 24H and 8H groups, with the degree increasing with the shortening of recovery (Fig. 6A, C).

### Oxidative stress

**Figure 7:**
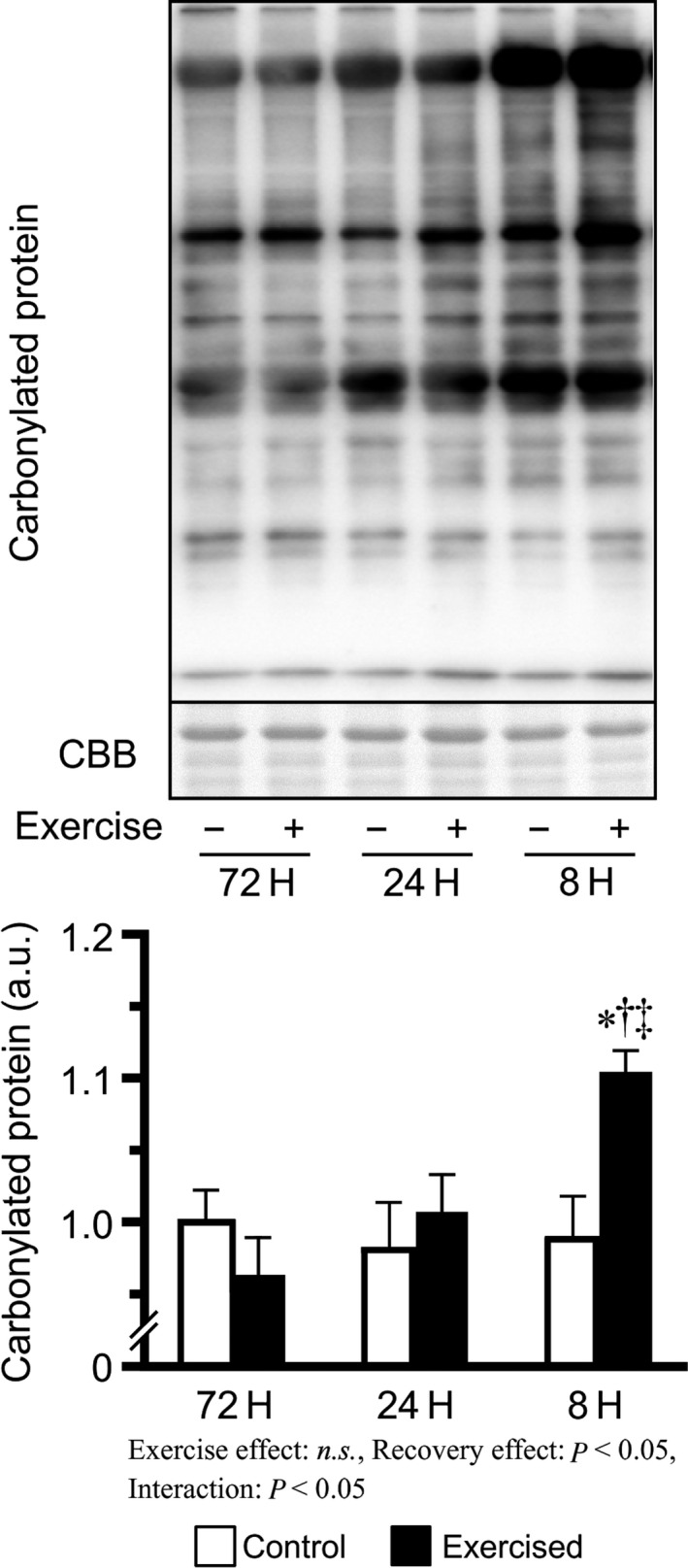
Carbonylated protein levels after bouts of resistance exercise in mouse skeletal muscle. Values are expressed relative to the no exercise 72H group and presented as the mean + SE. *P <* *0.05 versus control in each group, ^†^ *P *<* *0.05 versus exercised muscle in the 72H group, ^‡^ *P *<* *0.05 versus exercised muscle in the 24H group. n.s. indicates not significant.

We next focused on oxidative stress as another factor suppressing translation and measured the carbonylated protein levels as a marker of oxidative stress. After three bouts of resistance exercise, carbonylated protein levels were found to increase only in the 8H group when compared to those of the 72H and 24H groups (Fig. 7).

## Discussion

This study investigated the effects of recovery time between bouts on resistance exercise by varying the duration of the resting interval between three successive bouts of resistance exercise and observing the corresponding changes in muscle protein synthesis and other related factors. The main findings in this study are as follows: (1) repetition of resistance exercises with 72 h and 24 h recovery period, but not with 8 h recovery, activated protein synthesis; (2) mTOR signaling was more strongly activated in mouse groups subjected to repetition of resistance exercises with shorter recovery periods; and (3) mice subjected to excessively short recovery periods between bouts showed elevated levels of oxidative stress. These results suggest that repeated bouts of resistance exercise with excessively short recovery periods between bouts activate mTOR signaling, but not protein synthesis, could possibly be due to increased oxidative stress.

In this study, protein synthesis after three bouts of resistance exercise was observed to increase in the 72H and 24H groups, but not in the 8H group. As mentioned above, mTOR signaling is a major regulator of protein synthesis and is activated by resistance exercise. Our current results showed that three bouts of resistance exercise resulted in the phosphorylation of mTOR signaling‐related factors (Akt^Ser473^, p70S6K^Thr389^, and rpS6^Ser240/244^) in a manner dependent on shortening of the recovery period. Phosphorylation of mTOR signaling‐related factors has been shown to persist for more than 8 h (Ogasawara et al. 2016; West et al. 2016). Therefore, our findings indicate that repetition of resistance exercise with a shorter recovery period induces the accumulation of phosphorylated mTOR signaling factors owing to insufficient recovery time for dephosphorylation. Moreover, repetition of resistance exercise with shorter recovery leads to stronger activation of mTOR signaling than that with longer recovery, as previously reported by Coffey et al. (2007). In addition, these results indicate that the suppression of protein synthesis observed in the 8H group is not owing to the inhibition of mTOR signaling. Similarly, Kato et al. (2015) reported that 500 repetitions of eccentric contractions resulted in p70S6K^Thr389^ phosphorylation but did not induce protein synthesis in rat skeletal muscles. Therefore, levels of phosphorylated mTOR signaling‐related factors measured in this study may not be reliable indicators of protein synthesis. Further studies are required to determine the regulatory role of mTOR signaling in protein synthesis during resistance exercise and to elucidate the mechanisms underlying the suppression of protein synthesis with shortened recovery periods.

As previously mentioned, eIF‐2*α* can regulate translation in response to excess translational activity, which is mediated through the unfolded protein response (Ma and Hendershot 2003). A recent study showed that a single session of resistance exercise triggers the unfolded protein response within 48 h after the exercise (Ogborn et al. 2014). In this study, we suspected that eIF‐2*α* ^Ser51^ phosphorylation is the mechanism responsible for the observed inhibition of protein synthesis in the 8H group. However, levels of phosphorylated eIF‐2*α* ^Ser51^ did not change in all three groups. Ogborn et al. (2014) argued that the pathway of unfolded protein response including eIF‐2*α* is not readily activated by resistance exercise and showed that a single session of resistance exercise is insufficient to induce eIF‐2*α* phosphorylation. Therefore, the present results suggest that repeated bouts of resistance exercise do not readily induce eIF‐2*α* ^Ser51^ phosphorylation and that translation regulation by eIF‐2*α* is not involved in inhibiting protein synthesis after bouts of resistance exercise with excessively short recovery periods.

To explore other mechanisms potentially responsible for the suppression of protein synthesis by the repetition of resistance exercise with excessively short recovery period, we focused on 4E‐BP1. Like p70S6K, 4E‐BP1 is a major downstream target of mTOR and is a well‐known translational repressor that binds to eukaryotic initiation factor (eIF) 4E. 4E‐BP1 phosphorylation results in the dissociation of 4E‐BP1 from eIF‐4E, after which eIF‐4E initiates translation by assembling with eIF‐4G to form the eIF‐4F complex (Gingras et al. 2001). In this study, repeated bouts of resistance exercise phosphorylated 4E‐BP1^Thr37/46^, but no differences among recovery periods between bouts were observed, whereas the phosphorylation of p70S6K^Thr389^ were higher in groups subjected to shorter recovery periods. Similar findings were reported in several previous studies, in which the observed phosphorylation states of 4E‐BP1^Thr37/46^ and p70S6K^Thr389^ were not always consistent (Dreyer et al. 2006; Koopman et al. 2006; Deldicque et al. 2008; Moore et al. 2011). Therefore, 4E‐BP1^Thr37/46^ phosphorylation likely regulates protein synthesis through a mechanism different from p70S6K, as observed in mice subjected to bouts of resistance exercise with excessively short recovery periods. In this study, we also observed *γ*‐form ratio of 4E‐BP1 that migrated the slowest among the three isoforms, dissociated from eIF‐4E and promoted translation, as a marker of 4E‐BP1 activities. However, shorter recovery groups showed higher increase in *γ*‐form ratio. Therefore, it is likely that insufficient phosphorylation and migration of 4E‐BP1 is not responsible for the failure to activate protein synthesis in mice subjected to bouts of resistance exercise with very short recovery periods.

In this study, we also investigated the phosphorylation of AMPK. AMPK is a key sensor of cellular energy status, and known as the major downregulator of protein synthesis. In this study, AMPK phosphorylation was increased with the shortening of recovery. Therefore, the inhibition of protein synthesis observed in the excessively short recovery group may have been caused by the phosphorylation of AMPK. However, some previous studies also reported that inhibition of protein synthesis by AMPK is mediated by inactivation of mTOR (Bolster et al. 2002; Williamson et al. 2006; Ogasawara et al. 2014). In this study, the phosphorylation of mTOR, p70S6K, and rpS6 was not inhibited in shorter recovery groups. Therefore, the phosphorylation of AMPK may not have been involved in the inhibition of protein synthesis, at least through mTOR inhibition, in the excessively short interval group.

We also explored oxidative stress as a possible factor influencing protein synthesis. Oxidative stress is an imbalance in oxidant and antioxidant levels (Kregel and Zhang 2007). Previous studies have reported that long‐term resistance training increases antioxidant capacity and reduces oxidative stress after resistance exercise (Çakir‐Atabek et al. 2010; Scheffer et al. 2012). However, Haraguchi et al. (2011) reported that exhaustive resistance training increases oxidative stress levels in the skeletal muscles. Previous studies on myotubes and other cells have suggested that oxidative stress suppresses general translational activity and inhibits protein synthesis through mechanisms such as the inhibition of eIF formation and eIF‐2*α* phosphorylation, although it is unclear whether the suppressive effect is mediated by the inactivation of mTOR signaling (Pham et al. 2000; Patel et al. 2002; Shenton et al. 2006; Pierre et al. 2014). In this study, levels of carbonylated proteins, a major indicator of oxidative stress, were elevated only in the exercised 8H group. These results suggest that bouts of resistance exercise with excessively short recovery period lead to an exhausted state characterized by increased oxidative stress, which in turn may inhibit translational activity and protein synthesis. However, as mentioned above, phosphorylated eIF‐2*α* ^Ser51^ levels did not change among all treatment groups, suggesting that resistance exercise exerts a small effect on eIF‐2*α* ^Ser51^ phosphorylation (Ogborn et al. 2014). Further studies are required to elucidate the precise targets of oxidative stress related to the inhibition of protein synthesis after bouts of resistance exercise with excessively short recovery periods.

We did not investigate the changes in protein degradation, which is a limitation of this study. Protein degradation is another important factor affecting protein metabolism. Akt is a countermeasure of FoxO, a major mediator of protein degradation, and may have been influenced by the shortening of recovery. Additional studies evaluating protein degradation are required to understand the detailed changes in protein metabolism in response to the shortening of recovery. In conclusion, repeated bouts of resistance exercise with excessively short recovery periods effectively activated mTOR signaling, but did not induce protein synthesis. The discrepancy between mTOR signaling activity and protein synthesis might be caused by elevated oxidative stress, which inhibits translational activity. The present results suggest that repeating resistance exercise with excessively short recovery periods in the same muscle is not effective in achieving muscle hypertrophy, and planning an appropriate exercise program is necessary. Further studies are required to reveal the detailed mechanisms of the inhibition of protein synthesis in repetition of resistance exercises with excessively short recovery periods, and identify a method for increasing the effects of exercise as well as confirming the clinical responses in humans.

## Conflict of Interest

No conflicts of interest, financial or otherwise, are declared by the authors.
